# Melatonin Inhibits the Ferroptosis Pathway in Rat Bone Marrow Mesenchymal Stem Cells by Activating the PI3K/AKT/mTOR Signaling Axis to Attenuate Steroid-Induced Osteoporosis

**DOI:** 10.1155/2022/8223737

**Published:** 2022-08-18

**Authors:** Meng Li, Ning Yang, Li Hao, Wei Zhou, Lei Li, Lei Liu, Fang Yang, Lei Xu, Gang Yao, Chen Zhu, Wei Xu, Shiyuan Fang

**Affiliations:** ^1^Department of Orthopaedics, The First Affiliated Hospital of USTC, Division of Life Sciences and Medicine, University of Science and Technology of China, Lujiang Road No. 17, Hefei, 230001 Anhui, China; ^2^Department of Oncology, The First Affiliated Hospital of USTC, Division of Life Science and Medicine, University of Science and Technology of China, Lujiang Road No. 17, Hefei, 230001 Anhui, China; ^3^Department of Obstetrics and Gynecology, The Second Affiliated Hospital of Anhui Medical University, Hefei, 230022 Anhui, China

## Abstract

Steroid-induced osteoporosis (SIOP) is a form of secondary osteoporosis, but its specific mechanism remains unclear. Glucocorticoid (GC-)-induced death of osteoblasts and bone marrow mesenchymal stem cells (BMSCs) is an important factor in SIOP. Ferroptosis is an iron-dependent type of programmed cell death and can be induced by many factors. Herein, we aimed to explore whether GCs cause ferroptosis of BMSCs, identify pathways as possible therapeutic targets, and determine the underlying mechanisms of action. In this study, we used high-dose dexamethasone (DEX) to observe whether GCs induce ferroptosis of BMSCs. Additionally, we established a rat SIOP model and then assessed whether melatonin (MT) could inhibit the ferroptosis pathway to provide early protection against GC-induced SIOP and investigated the signaling pathways involved. *In vitro* experiments confirmed that DEX induces ferroptosis in BMSCs. MT significantly alleviates GC-induced ferroptosis of BMSCs. Pathway analysis showed that MT ameliorates ferroptosis by activating the phosphatidylinositol 3-kinase (PI3K)/protein kinase B (AKT)/mammalian target of rapamycin (mTOR) axis. MT upregulates the expression of PI3K, which is an important regulator of ferroptosis resistance. PI3K activators mimic the antiferroptotic effect of MT, but when the PI3K pathway is blocked, the effect of MT is weakened. Using *in vivo* experiments, we confirmed the in vitro results and observed that MT can obviously protect against SIOP induced by GC. Notably, even after the initiation of GC-induced ferroptosis, MT can confer protection against SIOP. Our research confirms that GC-induced ferroptosis is closely related to SIOP. MT can inhibit ferroptosis by activating the PI3K/AKT/mTOR signaling pathway, thereby inhibiting the occurrence of SIOP. Therefore, MT may be a novel agent for preventing and treating SIOP.

## 1. Introduction

Steroids are a large class of anti-inflammatory agents with therapeutic efficacy that are usually used for the treatment of inflammation-related syndromes [1]. However, the widespread use of these drugs has caused serious side effects. In recent decades, the prevalence of steroid-induced osteoporosis (SIOP) has increased rapidly, showing a trend toward younger patients [2–4]. Osteoporosis can lead to a substantial increase in the incidence of fragility fractures of the spine and extremities and increase treatment costs for patients [5]. However, there is no effective clinical strategy for the prevention and treatment of SIOP [6, 7].

The mechanism of SIOP induction by glucocorticoids (GCs) is unclear. However, the decreased number of osteoblasts is one of the main features of SIOP [8]. Bone marrow mesenchymal stem cells (BMSCs) are the main sources of osteoblasts in bones [9]. Studies have shown that the excessive use of steroids can cause oxidative stress in BMSCs. Under severe oxidative stress conditions, reactive oxygen species (ROS) accumulation in cells leads to increased endoplasmic reticulum stress, mitochondrial DNA (mtDNA) damage, mitochondrial dysfunction, and ultimately cell death [10].

Ferroptosis is a recently discovered form of programmed cell death caused by iron metabolism disorders and is closely related to various bone diseases [11, 12]. Unlike autophagy and necrosis, ferroptosis is characterized by inhibition of glutathione peroxidase (GPX)4 and system xc^−^, which leads to impairment of cysteine metabolism and enhanced lipid peroxidation. Ultimately, the promotion of the Fenton reaction leads to the production of a large amount of ROS and thus cell death [13, 14]. According to our previous report (Fang Shiyuan et al.), high-dose GC administration leads to downregulation of GPX4 expression in osteoblasts and enhancement of oxidative stress, indicating that GCs may induce ferroptotic effects and participate in pathogenic processes [15]. Therefore, we speculate that reducing the incidence of ferroptosis of BMSCs may have a positive effect on alleviating SIOP.

Melatonin (MT), synthesized from serotonin in the human pineal gland, performs several important functions, such as promoting bone formation and exerting antioxidative, anti-inflammatory, and antitumor effects [16, 17]. Several studies have shown that MT is an effective endogenous antioxidant that indirectly stimulates certain antioxidant enzymes, such as superoxide dismutase (SOD) and GPX [18–22]. In recent years, researchers have discovered that MT can ameliorate osteoporosis by inhibiting hyperglycemia-induced ferroptosis of osteoblasts [23]. However, whether MT can relieve SIOP by reversing the ferroptotic pathway in hormone-treated BMSCs and the specific mechanism remains unclear. To address the above issues, we established cellular and animal models of SIOP to evaluate the correlation between GCs and BMSCs ferroptosis and the role of MT in the treatment of SIOP.

## 2. Materials and Methods

### 2.1. Animal Studies

The animal protocol was approved by the Animal Ethics Committee of the First Affiliated Hospital of the University of Science and Technology of China (Hefei, China). Forty 8-week-old male Sprague-Dawley (SD) rats (weight: 300 ± 50 g) were obtained from the Experimental Animal Center of the University of Science and Technology of China and were randomly divided into four groups: (1) the control group (treated with saline only); (2) the SIOP model group (treated with 20 mg/kg DEX (i.m.) for eight weeks); (3) the MT pretreatment group (treated with 50 mg/kg MT (i.p.) (Sigma-Aldrich, USA) on the first day, 20 mg/kg DEX (i.m.) +50 mg/kg MT (i.p.) for eight weeks beginning on the second day, and then 50 mg/kg MT (i.p.) for four weeks); and (4) the MT posttreatment group (treated with 20 mg/kg DEX (i.m.) for eight weeks and then 50 mg/kg MT (i.p.) for four weeks). Finally, rats from each group were sacrificed under general anesthesia. Surgical instruments were used to remove muscle and soft tissue, and the entire femur was removed for subsequent experiments.

### 2.2. Microcomputed Tomography (micro-CT) Scans

To evaluate the changes in bone morphology in the rats, we performed high-resolution micro-CT (SKYSCAN1176, Aartselaar, Belgium) of the bilateral lateral femur with the following scan parameters: 18 *μ*m, 70 kV, and 141 mA. Subsequently, a CT analyzer (CTAN, Bruker) was used to analyze the images and perform 3D image reconstruction. Morphometric parameters were used to evaluate the bone mineral density (BMD mg/cm^3^), the bone surface/bone volume (BS/BV, %), the bone volume/total volume (BV/TV, %), the number of trabecular bones (Tb.N, 1/mm), the trabecular bone spacing (Tb.Sp, mm), and the trabecular bone thickness (Tb.Th, mm).

### 2.3. Hematoxylin and Eosin (H&E) Staining

H&E staining was performed to observe the changes in bone tissue structure and morphology. Femoral specimens were fixed in 4% paraformaldehyde for 24 h and decalcified with 10% ethylenediaminetetraacetic acid (EDTA, Sigma-Aldrich, USA) for 4 weeks. Thereafter, the decalcified femur samples were embedded in paraffin, cut into 7-*μ*m-thick sections, and placed on glass slides. Finally, the femoral sections were subjected to H&E staining. Briefly, the sections were dehydrated in ethanol, deparaffinized in xylene, stained with hematoxylin for 10 min, and then stained with eosin for 20 s. After rinsing, the samples were sealed with neutral resin. The staining results were observed under a laser scanning microscope (LSM, Carl Zeiss, Oberkochen, Germany).

### 2.4. Histological and Immunohistochemical Analysis

Fixation, decalcification, embedding, and slicing of rat femur specimens were performed as described above for H&E staining. Immunohistochemical analysis was then performed to further examine the localization and for qualitative and relative quantification of ferroptosis-related antigens in tissues. For immunohistochemical staining, the sections were deparaffinized, antigen repaired, and blocked with horse serum for 30 min. Then, to identify differences in the expression of ferroptosis markers in specimens from the four groups, the samples were incubated with system xc^−^ (1 : 500, orb100617, Biorbyt), ACSL4 (1 : 250, ab155282, Abcam), FSP1 (1 : 2000, ab197896, Abcam), and GPX4 (1 : 200, orb340797, Biorbyt) primary antibodies for 12 h followed by relevant biotinylated secondary antibodies. The staining results were observed under a laser scanning microscope.

### 2.5. Immunofluorescence Staining

To more fully locate and quantitatively examine antigenic substances in tissues, sections were first stained with primary antibodies against GPX4 (1 : 200, orb340797, Biorbyt), CD90 (1 : 500,RT1615, Huabio), and PI3K (1 : 500, orb621689, Biorbyt) for 12 h at 4°C. Afterwards, the sections were rinsed, and corresponding fluorescent secondary antibodies (Alexa Fluor 647- and Alexa Fluor 488-conjugated) were added for another 1 h of incubation in the dark. All sections were counterstained with 4,6-diamidine-2-phenylindole (DAPI) (Beyotime, China) and then observed under a fluorescence microscope (Axio Imager 2, Zeiss, Germany). The fluorescence intensity was assessed using ImageJ software (Bethesda, USA).

### 2.6. Cell Culture

BMSCs were obtained from the femurs and tibias of healthy 6-week-old SD rats. Freshly extracted BMSCs were cultured in *α* essential medium (*α*-MEM, HyClone, Logan, UT, USA) containing 10% fetal bovine serum (FBS) and 1% penicillin/streptomycin. Petri dishes containing BMSCs were placed in an incubator at 37°C in 5% CO_2_. Every three days, the medium was changed, and the BMSCs were subcultured. Cells were used at 3-6 passages in this study.

### 2.7. Cell Immunotyping and Characterization

Further studies were performed to assess the immunotyping and characterization in cells. After incubation with basic medium for 3 days, glass slides containing BMSCs were rinsed with PBS three times and fixed in ice-cold paraformaldehyde for 15 min. Next, the samples were incubated with QuickBlockTM IF blocking solution (Beyotime Biotech) for 1 h and incubated with primary CD90 (1 : 500, RT1615, Huabio) and CD105 (1 : 1000, PAB46055, Bioswamp) antibodies at 4°C overnight. After incubation, the samples were rinsed three times with PBS and incubated with secondary antibody at 37°C for 1 h. Finally, DAPI was used to stain the cell nuclei, and the results were imaged using a laser scanning microscope.

### 2.8. Validation of Multidirectional Differentiation of Cells

For osteogenic differentiation, BMSCs were cultured on glass slides for 3 days, and then, the culture medium was refreshed with the osteogenic medium (DMEM supplemented with 0.1 *μ*M dexamethasone, 50 *μ*M ascorbic acid, 10 mM *β*-glycerol phosphate, and 10% FBS) for osteogenic culture. After 7 days of osteogenic induction, the BMSCs were determined by ALP staining. In particular, the cells after 7 days of osteogenic induction were rinsed with PBS for 3 times, fixed with paraformaldehyde (4 wt. %) at 4°C for 30 minutes, and then incubated in a 5-bromo-4-chloro-indolyl phosphate/nitroblue tetrazolium chloride (BCIP/NBT) working solution (Beyotime, China) in the dark for 20 min. After 21 days of osteogenic culture, the cells cultured on glass slides were fixed with 95% ethanol for 15 minutes, and 40 mM Alizarin red S (ARS, Beyotime, China) was added for staining at room temperature for 30 minutes. Subsequently, the cells were washed 3 times with deionized water and observed by optical microscopy (DM750M, Leica, Germany).

For adipogenic differentiation, BMSCs cultured on glass slides were incubated with adipogenic differentiation medium for 2 weeks. Afterwards, the cells were washed 3 times with PBS and then fixed in 4% paraformaldehyde for 20 min. The cells were washed 3 times with ddH_2_O, followed by five-minute incubation in 60% isopropanol. Next, the cells were incubated in Oil Red O (Sigma, USA) solution for 10 min and then were rinsed with 60% isopropanol and ddH_2_O again. Finally, the stained cells were observed and captured using a laser microscope.

### 2.9. Cell Proliferation and Toxicity Assay

A cell counting kit (CCK-8) (Beyotime, China) was used to determine the cytotoxicity of DEX and MT. Briefly, BMSCs were cultured at a density of 5 × 10^3^ cells/well in a 96-well plate. The cells were then incubated with various concentrations of DEX (10^−5^, 10^−4^, 10^−3^, and 10^−2^ M) or MT (0, 5, 10, 50, 100, and 200 *μ*M) for 24, 48, or 72 h. To evaluate BMSCs activity, we added a solution containing 90% serum-free *α*-MEM and 10% CCK-8 to each well and then cultured the cells for 2 h at 37°C in the dark. The absorbance of each well was measured at 450 nm using a microplate reader (BioTek, Vermont, USA).

### 2.10. Cell Death Assay

To assess the extent of cell death, BMSCs were cultured in three replicate wells of a 24-well plate (cell density: 1 × 10^4^ cells per well) for 72 h, with three replicate wells being included for each group. An Annexin V-mCherry/SYTOX Green detection kit (C070M, Beyotime, China) was used to assess cell death. The BMSCs were incubated with DEX (10^−4^, 10^−3^, and 10^−2^ M) and different doses of MT (10 *μ*M and 100 *μ*M) for 24 h. The cells were then incubated with medium containing 194 *μ*L Annexin V-mCherry binding buffer, 5 *μ*L Annexin V-mCherry, and 1 *μ*L SYTOX Green for 20 min at 37°C in the dark. A laser scanning microscope was used to image the results and determine the level of cell death.

### 2.11. ROS Assay

To examine changes in oxidative stress levels during ferroptosis, cells were cultured in 24-well plates (cell density: 1 × 10^4^ cells/well) for 72 h, with three replicate wells being included for each group. To measure the levels of intracellular ROS, BMSCs were incubated with *α*-MEM medium containing a gradient of DEX (10^−4^, 10^−3^, and 10^−2^ M) or 10^−3^ M DEX supplemented and different doses of MT (10 and 100 *μ*M) for 24 h. The cells were incubated with medium containing 2,7-dichlorofluorescein diacetate (DCFH-DA, 1 : 1,000 dilution) at 37°C for 20 min in the dark. After three washes in phosphate-buffered saline (PBS), an Olympus IX51 microscope was used to image the results and determine ROS levels. ROS content was determined based on the average fluorescence intensity.

### 2.12. Immunofluorescence Assay

Further studies were performed to assess the localization and the amounts of ferroptosis-related antigens in cells. After incubation with medium without DEX or medium supplemented with various doses of DEX and MT, glass slides containing BMSCs were rinsed with PBS three times and fixed in ice-cold paraformaldehyde for 15 min. Next, the samples were incubated with QuickBlockTM IF blocking solution (Beyotime Biotech) for 1 h and incubated with primary GPX4 (1 : 200, orb340797, Biorbyt) and PI3K (1 : 500, orb621689, Biorbyt) antibodies at 4°C overnight. After incubation, the samples were rinsed three times with PBS and incubated with fluorescein isothiocyanate secondary antibody at 37°C for 1 h. Finally, DAPI was used to stain the cell nuclei, and the results were imaged using a laser scanning microscope.

### 2.13. Enzyme-Linked Immunosorbent Assay (ELISA)

The MT protein level was quantified using an ELISA. Two milliliters of blood was collected from rats in each group, and EDTA was used for anticoagulation. Serum samples were collected by centrifuging the blood samples at 1000 rpm for 20 min at 4°C. An MT ELISA kit (Abbexa, Cambridge, UK) was used according to the manufacturer's instructions, and the absorbance was measured at 490 nm or 540 nm using an ELX800 microplate reader (Bio-Tec, USA).

### 2.14. Quantitative Reverse Transcription-Polymerase Chain Reaction (qRT-PCR)

The expression levels of relevant genes were detected during ferroptosis. After cell treatment, TRIzol reagent (Beyotime, China) was added to extract total RNA from BMSCs in the different groups, and the RNA was reverse transcribed into cDNA using a synthesis kit (Takara Bio, Japan). Using a mixture of dNTP reagent (Takara, Japan) and RNase-free H_2_O (Abcam, Cambridge, UK), the levels of the system xc^−^, GPX4, ACSL4, and FSP1 genes were analyzed using qRT-PCR (S1000, Bio-Rad, USA). Similarly, for PCR analysis of bone tissue, full-length bone tissue was collected from the femur, and the bone marrow cavity was washed with ice-cold PBS. The bone tissue was chopped and ground into a powder in liquid nitrogen. Before the liquid nitrogen had evaporated, the powder was transferred to a centrifuge tube, and total RNA (2 *μ*g) was extracted using a TRIzol kit (Beyotime, China) according to standard operating procedures. The rest of the procedure was performed as described above for cell detection. The 2^-*ΔΔ*CT^ method was used to analyze the gene expression data, and the glyceraldehyde phosphate dehydrogenase (GAPDH) gene was used as a housekeeping gene for normalization.

*System xc* ^−^ forward: 5′-ATACGCTGAGTGTGGTTTGC-3′

*System xc^−^* reverse: 5′-CTTCATCCACTTCCACAGCG-3′

*GPX4* forward: 5′-ATACGCTGAGTGTGGTTTGC-3′

*GPX4* reverse: 5′-CTTCATCCACTTCCACAGCG-3′

*ACSL4* forward: 5′-TGAATGTCTGCTTCTGCTGC-3′

*ACSL4* reverse: 5′-CCAACTCTTCCAGTAGTGTAGTCGG-3′

*FSP1* forward: 5′-AGCTTCTTGGGGAAAAGGAC-3′

*FSP1* reverse: 5′-CCCCAACCACAT CAGAGG-3′

*GAPDH* forward: 5′-TGACCTCAACTACATGGTCTACA-3′

*GAPDH* reverse: 5′-CTTCCCATTCTCGGCCTTGTACA-3′

### 2.15. Western Blotting

After subsequent processing, BMSCs were harvested, and proteins were extracted using radioimmunoprecipitation assay (RIPA) buffer (NCM Biotech, Soochow, China). Protein was obtained by collecting the supernatant after centrifugation (14,800*×g*) for 25 min at 4°C. After the total protein concentration of protein in each group was determined and standardized, equal amounts of the protein samples were separated using 10% sodium dodecyl sulfate (SDS)-polyacrylamide gel electrophoresis (PAGE) (Beyotime Biotech) and transferred onto nitrocellulose membranes. After blocking nonspecific binding sites using QuickBlock Blocking Buffer (Beyotime Biotech) for 1 h, the membranes were incubated with primary antibodies overnight at 4°C. The primary antibodies included system xc^−^ (1 : 2,000, orb100617, Biorbyt), ACSL4 (1 : 10,000, ab155282, Abcam), FSP1 (1 : 1,000, ab197896, Abcam), GPX4 (1 : 2,000, orb340797, Biorbyt), PI3K-p85 (1 : 1,000, orb621689, Biorbyt), AKT (1 : 1,000, orb99431, Biorbyt), p-ATK (1 : 1,000, orb344403, Biorbyt), mTOR (1 : 2,000, orb99435, Biorbyt), and p-mTOR (1 : 1,000, orb315804, Biorbyt) antibodies. Thereafter, the membranes were incubated with corresponding secondary antibodies (1 : 5,000) for 1 h. Finally, we used enhanced chemiluminescence (Pierce ECL) to visualize the protein bands, and the relative gray values were measured using Image Lab 3.0. Similarly, for Western blot analysis of bone tissue, full-length femur bone tissue was collected, and the bone marrow cavity was flushed with ice-cold PBS. The bone tissue was chopped and ground into a powder in liquid nitrogen. Before the liquid nitrogen had volatilized, the powder was transferred to a centrifuge tube, and RIPA buffer (NCM Biotech, Soochow, China) was added at a ratio of 1 : 10. After lysis and homogenization, the samples were incubated on ice for 40 min and centrifuged at 12, 000×*g* at 4°C for 15 min. The supernatant, which was the total bone protein, was collected. The remaining steps were performed as described above for cell detection.

### 2.16. Statistical Analysis

SPSS statistical software version 20 was used for analysis, and all results are expressed as the mean ± standard deviation. One-way analysis of variance (ANOVA) was used to determine the statistical significance. All post hoc analyses were conducted using Tukey's test. Statistical significance was set as *P* < 0.05.

## 3. Results

### 3.1. DEX Activates the Ferroptosis Pathway in BMSCs

**Figure 1:**
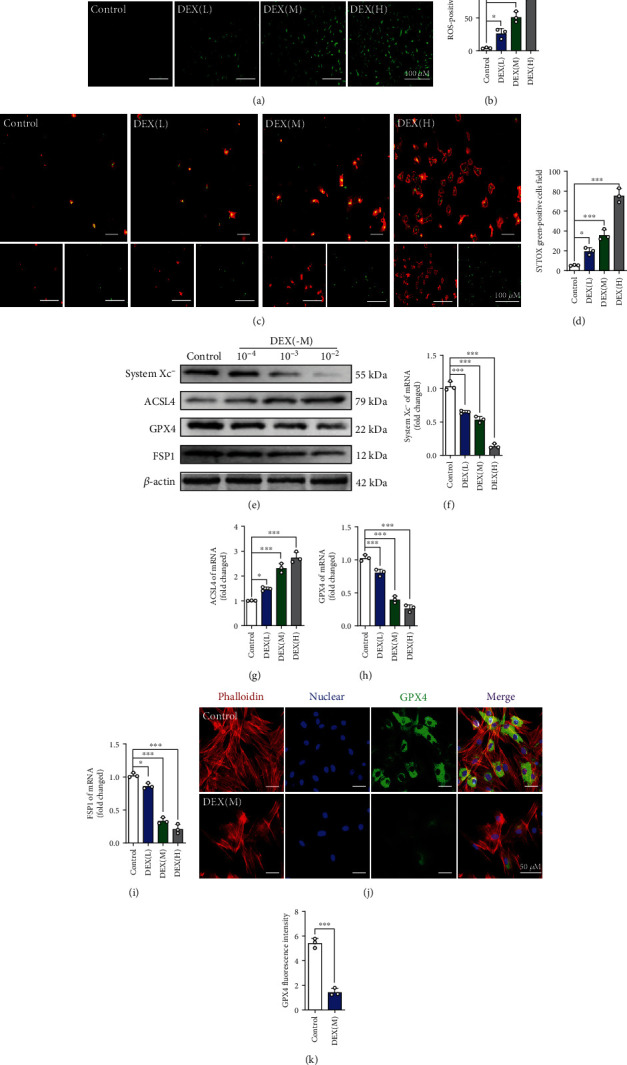
DEX activates the ferroptotic pathway of BMSCs. (a) ROS staining was performed to test the correlation between different concentrations of DEX and the level of oxidative stress. (b) Quantitative analysis of the number of ROS-positive cells per field in (a). (c) Annexin V-mCherry/SYTOX Green detection kit was used to detect cell death. (d) Quantitative analysis of the percentage of SYTOX green-positive cells in (c). (e–i) BMSCs were treated with various concentrations of DEX for 24 h, and the expressions of system xc-, ACSL4, GPX4, and FSP1were analyzed by western blot and qRT-PCR. (j) Images of immunofluorescence staining of GPX4 in BMSCs after treatment with DEX (10^−3^ M) for 72 h. (k) Quantification of the fluorescence intensity of GPX4 immunofluorescence positively stained cells. These studies were performed at least 3 biological replicates. Data represent mean ± SD (*n* = 3). ^∗^*P* < 0.05, ^∗∗^*P* < 0.01, ^∗∗∗^*P* < 0.005 compared with control group.

**Figure 2:**
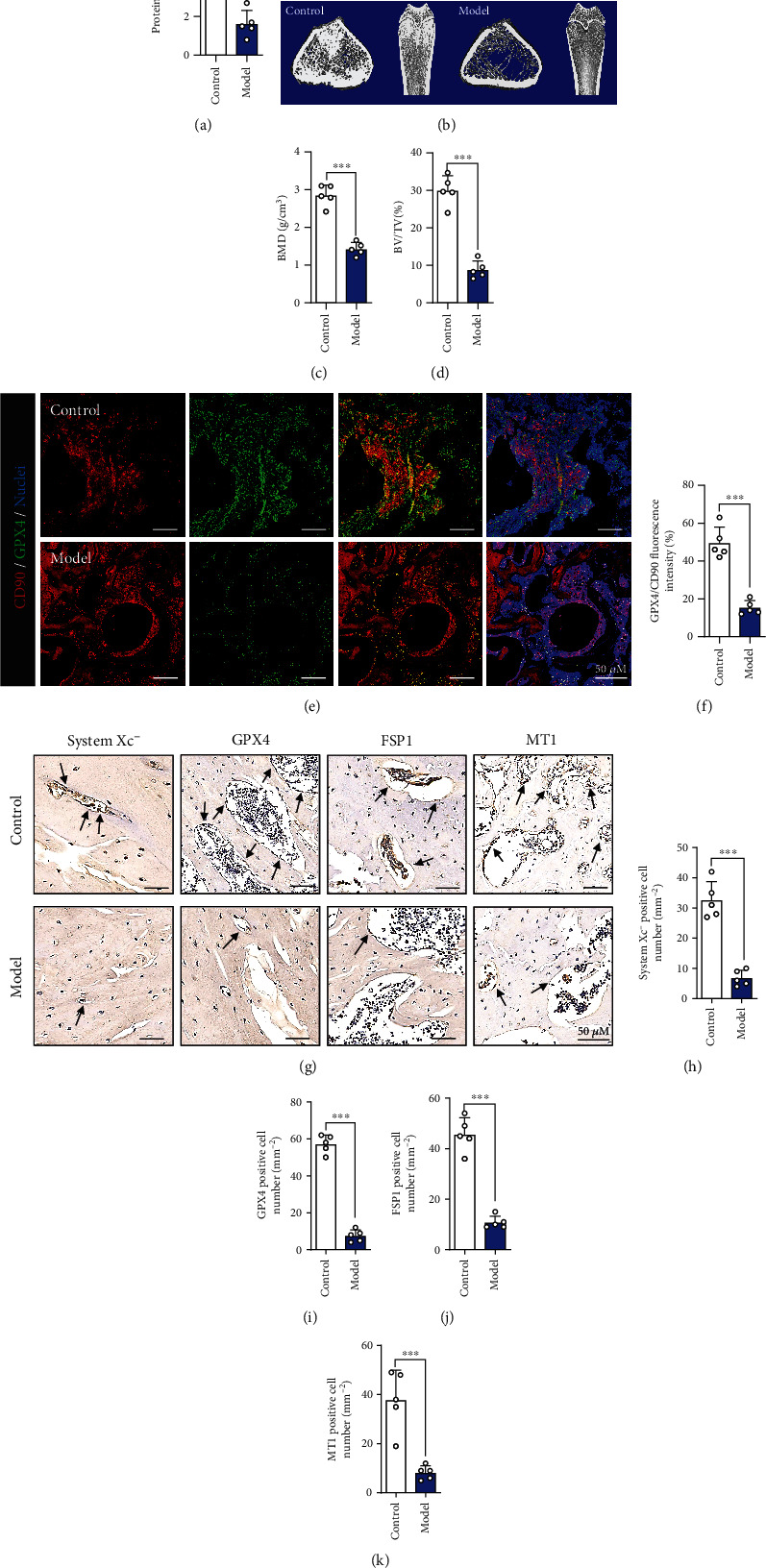
DEX activates the ferroptotic pathway in SIOP. (a) Quantification of the protein level of MT by ELISA. (b) Images of micro-CT. (c) BMD(g/cm^3^). (d) BV/TV (%). (e) Images of immunofluorescence double staining of CD90 and GPX4 in bone tissues. (f) Quantitative analysis of the area of GPX4/CD90-positive stains. (g) IHC staining of system xc-, GPX4, FSP1, and MT1, and the IHC-positive cells were marked with black arrows. (h–k) Quantitative analysis of the number of the IHC-positive cells in (g). These studies were performed at least 3 biological replicates. Data represent mean ± SD (*n* = 5). ^∗^*P* < 0.05, ^∗∗^*P* < 0.01, ^∗∗∗^*P* < 0.005 compared with control group.

Prior to the cell experiments, the BMSCs used in this study were characterized for the surface markers and examined for the multilineage differentiation potential after osteogenic and adipogenic differentiation. As shown in Figure S1, the cells used in this study express the specific surface molecules of BMSCs as CD90 and CD105, and provide the potential of multi-directional differentiation, which verified that mesenchymal stem cells were used. The results of the CCK-8 assay showed that DEX at concentrations ranging from 10^−5^ M to 10^−2^ M had a significant inhibitory effect on the proliferation of BMSCs (Figure S2a). We used oxidative stress kits and cell death kits to confirm whether DEX affected the ferroptosis pathway of BMSCs. The results showed that as the DEX concentration increased, the ROS levels in BMSCs increased, especially at DEX concentrations of 10^–3^ M and 10^–2^ M **(**Figures 1(a) and 1(b)**)**. Furthermore, the number of dead BMSCs was significantly increased after DEX treatment **(**Figures 1(c) and 1(d)**)**. Additionally, as the concentration of DEX increased, the levels of ferroptosis-related indicators system xc^−^, ACSL4, GPX4, and FSP1 were altered to varying degrees **(**Figures 1(e)–1(i)**)**. Cellular immunofluorescence staining also confirmed that GPX4 was expressed in the cytoplasm of BMSCs. We found that the expression of GPX4 in BMSCs was significantly downregulated after DEX intervention **(**Figures 1(j) and 1(k)**)**. Furthermore, the results indicated that the level of ferroptosis induced by DEX was positively correlated with stimulation time (Figures S3a–i). To explore whether MT is associated with SIOP, an ELISA kit was used to determine the expression of MT receptors in BMSCs. The results showed that the expression of MT decreased with increasing DEX concentration **(**Figure 2(a)**)**. To verify these *in vitro* results, we obtained femoral tissues from the SIOP model group after treatment with DEX (20 mg/kg) for eight weeks and the control group for comparison (Figure S4a). Micro-CT images and computed tomography (CT) analysis showed that trabeculae were sparse and thinner and showed wider gaps in the model group than in the control group and that the trabecular arrangement was disordered in the model group **(**Figures 2(b)–2(d)**)**. Double staining of a ferroptosis-related marker (GPX4) and a BMSC marker (CD90) was performed **(**Figures 2(e)–2(f)**)**, and the results showed that DEX treatment induced BMSC ferroptosis. Ferroptosis-related indicators were detected using immunohistochemical staining. We found that in the SIOP model group, the number of positive cells was significantly decreased **(**Figures 2(g)–2(k)**)**. The expression of system xc^−^, GPX4, FSP1, and MT1 in bone tissue was downregulated to varying degrees, while ACSL4 expression was upregulated (Figures S4b–g). Therefore, these data indicate that DEX induces ferroptosis in a rat SIOP model.

### 3.2. A Ferroptosis Inhibitor Reduces DEX-Induced Cell Death in BMSCs

**Figure 3:**
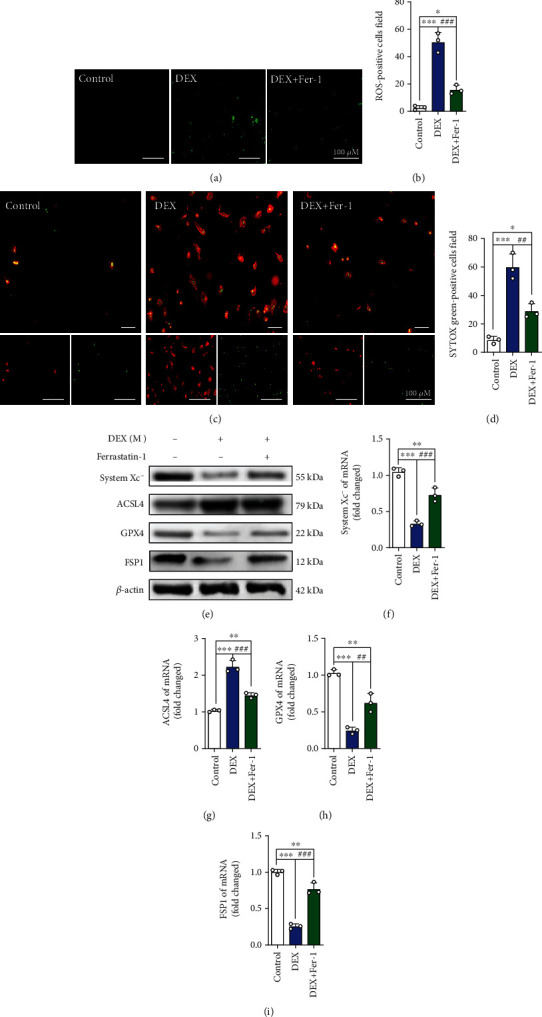
Ferroptosis inhibitor regulates the expression of related proteins in BMSCs. (a) ROS staining was performed to test the level of oxidative stress in control group, DEX (10^−3^ M) group and DEX + Fer-1 group. (b) Quantitative analysis of the number of ROS-positive cells per field in (a). (c) Annexin V-mCherry/SYTOX Green detection kit was used to detect cell death. (d) Quantitative analysis of the percentage of SYTOX green-positive cells in (c). (e–i) Western blot and qRT-PCR results for the expressions of system xc-, ACSL4, GPX4, and FSP1 were pretreated with ferroptosis inhibitor Ferrastatin-1 for 24 h; DEX (10^−3^ M) was then added for 24 h. These studies were performed at least 3 biological replicates. Data represent mean ± SD (*n* = 3). ^∗^*P* < 0.05, ^∗∗^*P* < 0.01, ^∗∗∗^*P* < 0.005 compared with control group. #*P* < 0.05, ##*P* < 0.01, ###*P* < 0.005 compared with DEX group.

To further verify whether ferroptosis is one of the main factors of DEX-induced BMSCs death, we exposed cells to DEX combined with a ferroptosis inhibitor (ferrastatin-1, Sigma-Aldrich, USA) and assessed ROS levels and cell death. The results showed that the ferroptosis inhibitor significantly decreased both the ROS levels and the number of dead cells **(**Figures 3(a)–3(d)**)**. Western blotting and qRT-PCR were used to evaluate the expression of the ferroptosis-related indicators system xc^−^, ACSL4, GPX4, and FSP1. We found that DEX altered the levels of system xc^−^, ACSL4, GPX4, and FSP1 to varying degrees but that ferrastatin-1 reversed this change to a certain extent **(**Figures 3(e)–3(i)**)**. Therefore, these results indicate that DEX-induced ferroptosis is the main cause of BMSCs death.

### 3.3. MT Reduces Ferroptosis in BMSCs Treated with DEX

**Figure 4:**
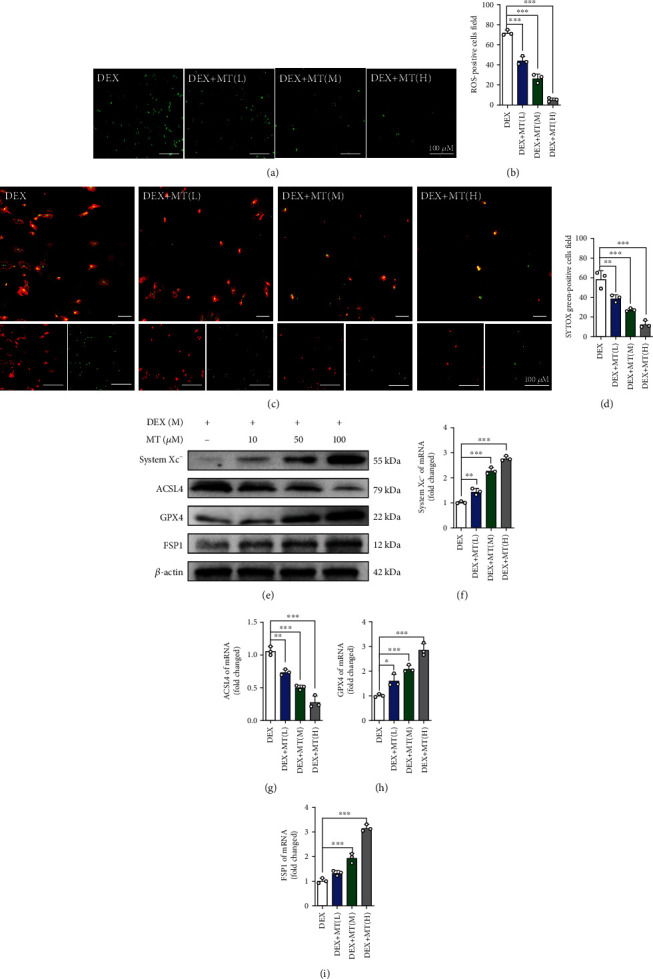
MT can reduce the ferroptosis level in BMSCs induced by DEX. (a) ROS staining was performed to test the correlation between different concentrations of MT and the level of oxidative stress. (b) Quantitative analysis of the number of ROS-positive cells per field in (a). (c) Annexin V-mCherry/SYTOX Green detection kit was used to detect cell death. (d) Quantitative analysis of the percentage of SYTOX green-positive cells in (c). (e–i) Western blot and qRT-PCR results for the expressions of system xc-, ACSL4, GPX4, and FSP1 were pretreated with various concentrations of MT for 24 h; DEX (10^−3^ M) was then added for 24 h. These studies were performed at least 3 biological replicates. Data represent mean ± SD (*n* = 3). ^∗^*P* < 0.05, ^∗∗^*P* < 0.01, ^∗∗∗^*P* < 0.005 compared with DEX group.

We investigated whether MT could inhibit the ferroptosis pathway in BMSCs. The CCK-8 assay results showed that at 5 *μ*M to 200 *μ*M, MT had no obvious toxic effects on BMSCs (Figure S2b). Then, BMSCs were incubated with DEX at a concentration of 10^−3^ M or DEX in addition to various MT concentrations (10, 50, and 100 *μ*M) for 24 h. ROS staining showed that low-dose (10 *μ*M), medium-dose (50 *μ*M), and high-dose (100 *μ*M) MT significantly reduced ROS levels in BMSCs, especially in the presence of at high doses of MT (Figures 4(a) and 4(b)). In addition, a cell death staining kit revealed that high doses of MT significantly reduced the number of dead cells (Figures 4(c) and 4(d)). Western blotting and qRT-PCR further confirmed that MT altered the levels of ferroptosis pathway-related indicators in BMSCs; increased the expression of system xc^−^, GPX4, and FSP1; and downregulated the expression of ACSL4 (Figures 4(e)–4(i)). These results indicate that MT inhibits DEX-induced ferroptosis in BMSCs.

### 3.4. MT Reduces Ferroptosis in DEX-Treated BMSCs by Activating the PI3K/AKT/mTOR Pathway

**Figure 5:**
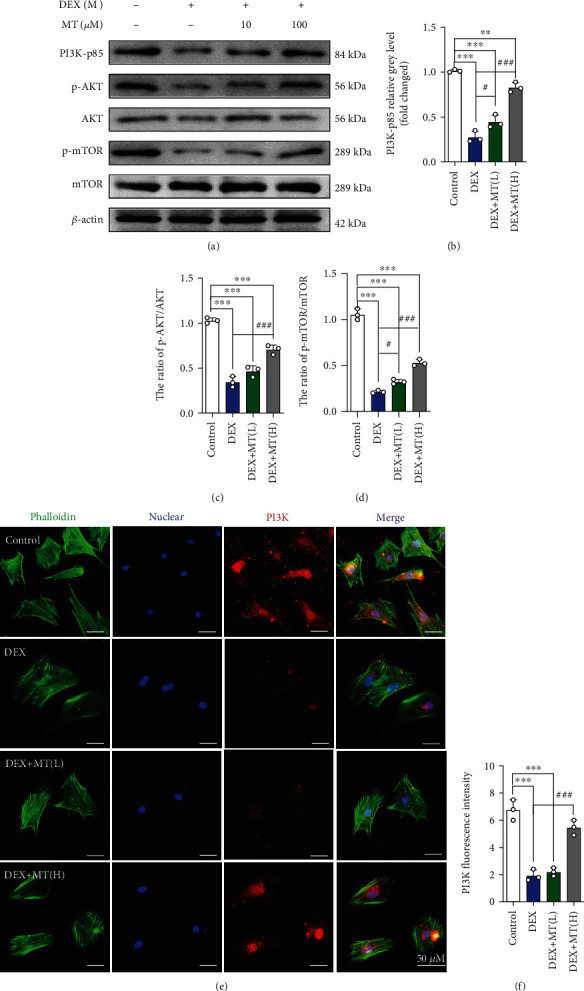
MT reduces ferroptosis in DEX-induced BMSCs by activating the PI3K-AKT-mTOR pathway. (a–d) Western blot results for the expressions of PI3K-p85, p-AKT, AKT, p-mTOR, and mTOR were pretreated with various concentrations of MT for 24 h; DEX (10^−3^ M) was then added for 24 h. (e) Images of immunofluorescence staining of PI3K in BMSCs. (f) Quantification of the fluorescence intensity of PI3K immunofluorescence positively stained cells. These studies were performed at least 3 biological replicates. Data represent mean ± SD (*n* = 3). ^∗^*P* < 0.05, ^∗∗^*P* < 0.01, ^∗∗∗^*P* < 0.005 compared with control group. #*P* < 0.05, ##*P* < 0.01, ###*P* < 0.005 compared with DEX group.

**Figure 6:**
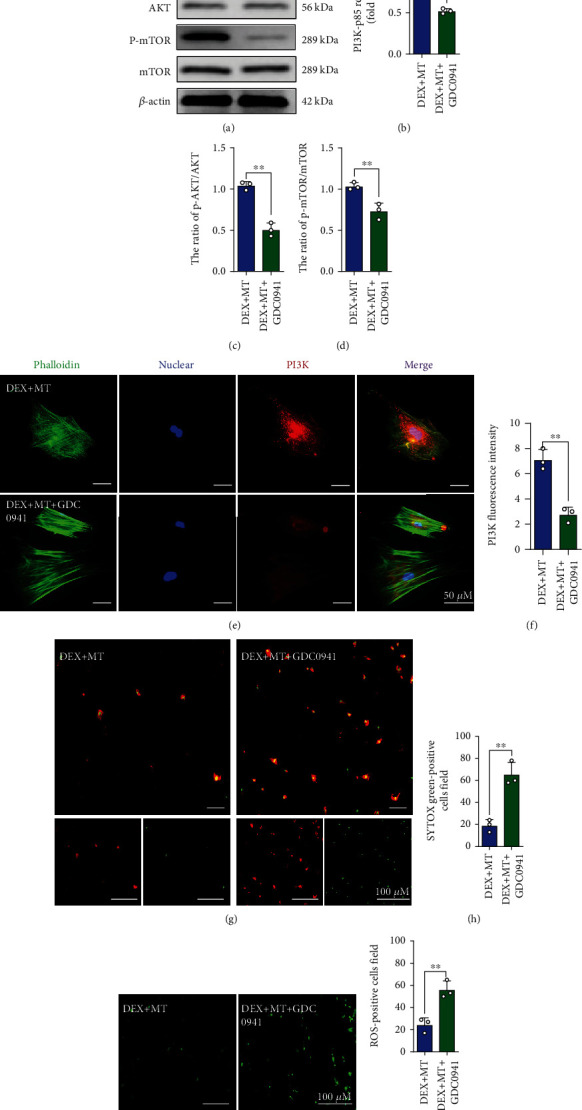
The positive regulatory effect of MT was significantly weakened with the use of PI3K inhibitors. (a–d) Western blot results for the expressions of PI3K-p85, p-AKT, AKT, p-mTOR, and mTOR were pretreated with GDC-0941 and MT for 24 h; DEX (10^−3^ M) was then added for 24 h. (e) Images of immunofluorescence staining of PI3K in BMSCs. (f) Quantification of the fluorescence intensity of PI3K immunofluorescence positively stained cells. (g) Annexin V-mCherry/SYTOX Green detection kit was used to detect cell death. (h) Quantitative analysis of the percentage of SYTOX green-positive cells in (g). (i) ROS staining was performed to test the level of oxidative stress in DEX + MT group and DEX + MT + GDC0941 group. (j) Quantitative analysis of the number of ROS-positive cells per field in (i). These studies were performed at least 3 biological replicates. Data represent mean ± SD (*n* = 3). ^∗^*P* < 0.05, ^∗∗^*P* < 0.01, ^∗∗∗^*P* < 0.005 compared with DEX + MT group.

To further understand the mechanism underlying the effect of MT on ferroptosis signaling, we exposed BMSCs to a concentration gradient of MT and observed changes in the expression of proteins related to the PI3K/AKT/mTOR pathway. As shown in Figures 5(a)–5(d), the western blot results show that DEX significantly downregulated the protein expression of PI3K-p85, p-AKT, and p-mTOR. However, when MT was applied, the expression of PI3K-p85, p-AKT, and p-mTOR appeared to be upregulated as the concentration increased. Furthermore, immunofluorescence showed that the addition of DEX significantly reduced the fluorescence intensity of PI3K. After MT treatment, the fluorescence intensity of PI3K in BMSCs was significantly enhanced **(**Figures 5(e) and 5(f)**)**. These results further indicate that MT activates the PI3K/AKT/mTOR pathway. We then investigated whether intervention with the PI3K pathway regulates GC-induced ferroptosis. The effect of a PI3K agonist (740 Y-P, 10 *μ*M, MedChemExpress, USA) in BMSCs was observed. As shown in Figures S5a–j, upon activation of PI3K in BMSCs, ROS levels are significantly reduced, and ferroptosis is significantly inhibited. 740 Y-P further enhanced the therapeutic effect of MT. Furthermore, GDC0941 (a selective inhibitor of PI3K, LC Laboratories, USA) was used to inhibit the expression of PI3K. As shown in Figures 6(a)–6(f), the expression of PI3K-p85, p-AKT, and p-mTOR is downregulated after GDC0941 was added. Western blot analysis showed that GDC0941 partially reversed the therapeutic effect of MT. ROS staining and a cell death assay showed that PI3K inhibitors significantly weakened the positive regulatory effect of MT and significantly increased the number of dead cells **(**Figures 6(g)–6(j)**)**. These analyses indicate that MT can reduce the level of ferroptosis and oxidative stress in DEX-induced BMSCs by activating the PI3K/AKT/mTOR pathway.

### 3.5. MT Attenuates Ferroptosis and Osteoporosis in a Rat SIOP Model

**Figure 7:**
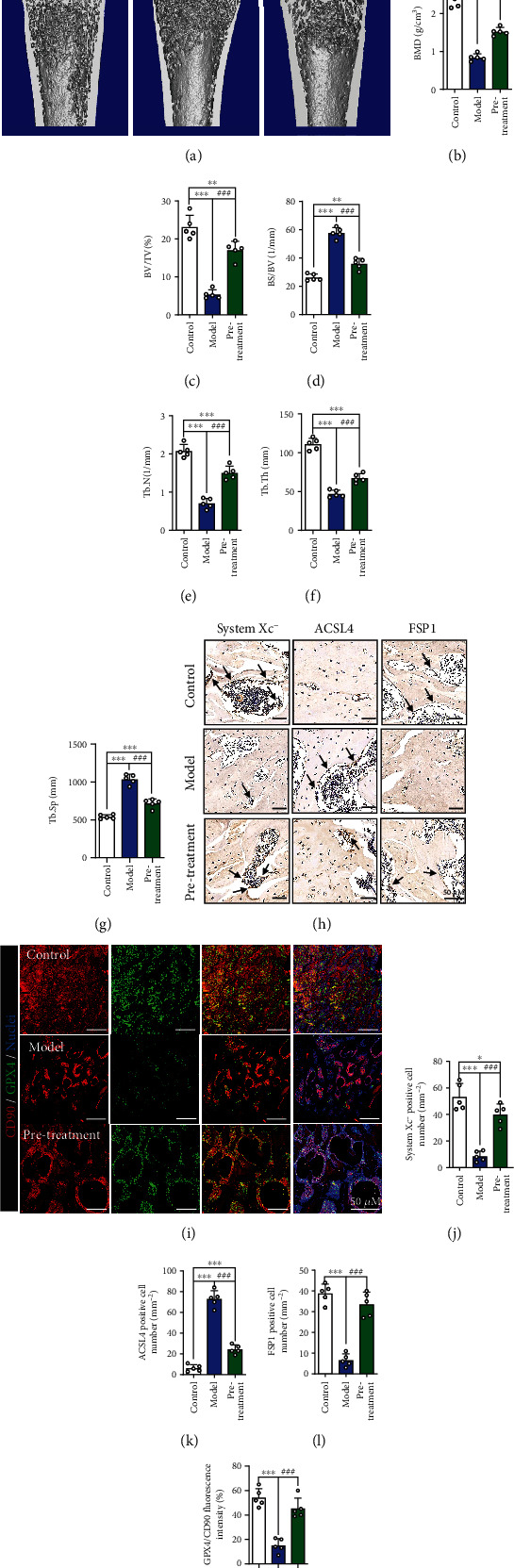
Pretreatment with MT alleviates DEX-induced SIOP in vivo. (a) Images of micro-CT. (b) BMD (g/cm^3^). (c) BV/TV (%). (d) BS/BV(1/mm). (e) Tb.N(1/mm). (f) Tb. Th (mm). (g) Tb. Sp (mm). (h) IHC staining of system xc-, ACSL4 and FSP1, and the IHC-positive cells were marked with black arrows. (i) Images of immunofluorescence double staining of CD90 and GPX4 in bone tissues. (j–l) Quantitative analysis of the number of the IHC-positive cells in (h). (m) Quantitative analysis of the area of GPX4/CD90-positive stains in (i). These studies were performed at least 3 biological replicates. Data represent mean ± SD (*n* = 5). ^∗^*P* < 0.05, ^∗∗^*P* < 0.01, ^∗∗∗^*P* < 0.005 compared with control group. #*P* < 0.05, ##*P* < 0.01, ###*P* < 0.005 compared with model group.

We further verified whether MT affects bone morphology in the early stages of SIOP *in vivo*. Figure S6a illustrates the timeline of DEX-induced SIOP model establishment and MT administration *in vivo*. The micro-CT images showed that the change of bone density was partially stopped, the bone trabeculae were thicker, and the trabecular arrangement was more regular in the pretreatment group **(**Figure 7(a)**)**. CT analysis further confirmed that the BMD, BV/TV, Tb. N, and Tb. Th values were significantly increased and the BS/BV and Tb. S. *P* values were significantly reduced in the MT pretreatment group compared with the model group **(**Figures 7(b)–7(g)**)**. In Figures 7(h) and 7(j)–7(l), the immunohistochemical staining results show that MT increased the number of cells positive for ferroptosis marker proteins such as system xc- and FSP1 and decreased those positive for ACSL4 in trabecular bone surfaces and the medullary cavities. The fluorescence intensity of PI3K was decreased in the model group compared with the control group, but MT pretreatment significantly stopped these changes (Figures S6g and i). In addition, H&E counterstaining after immunofluorescence staining was performed to observe the microstructural changes in bone tissue and the localization of GPX4 in vivo (Figures 7(i) and 7(m); Figures S6h, j), and the results showed that MT pretreatment increased the expression of GPX4 in BMSCs. Western blotting and qRT-PCR were used to assess the expression of the ferroptosis pathway-related indicators system xc^−^, ACSL4, GPX4, and FSP1 in bone tissues from the three groups. We found that, compared with model group, the expression of system xc^−^, GPX4, and FSP1 in the pretreatment group was upregulated to varying degrees and that ACSL4 expression was downregulated (Figures S6b–f). These results indicate that MT significantly decreases ferroptosis and ameliorates osteoporosis in an SIOP model.

### 3.6. MT Can Improve SIOP after the Initiation of DEX-Induced Ferroptosis

**Figure 8:**
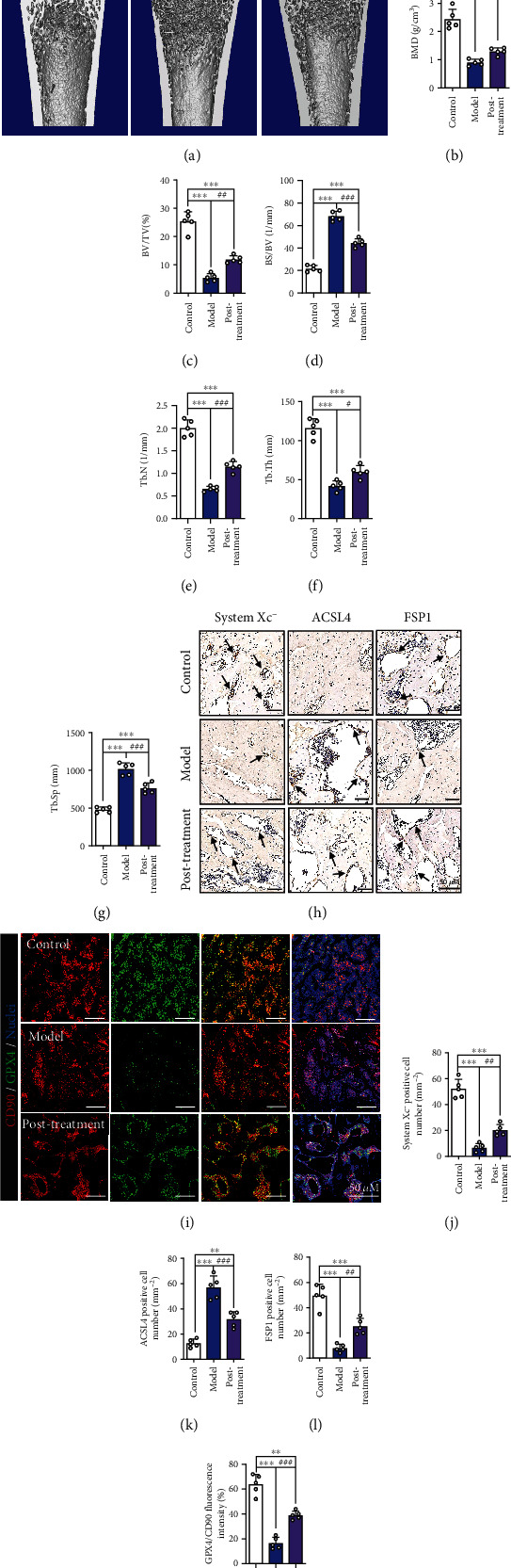
Even after DEX-induced ferroptotic effects begin, MT can still improve SIOP. (a) Images of micro-CT. (b) BMD (g/cm^3^). (c) BV/TV (%). (d) BS/BV (1/mm). (e) Tb.N (1/mm). (f) Tb.Th (mm). (g) Tb.Sp (mm). (h) IHC staining of system xc-, ACSL4, and FSP1, the IHC-positive cells were marked with black arrows. (i) Images of immunofluorescence double staining of CD90 and GPX4 in bone tissues. (j–l) Quantitative analysis of the number of the IHC-positive cells in (h). (m) Quantitative analysis of the area of GPX4/CD90-positive stains in (i). These studies were performed at least 3 biological replicates. Data represent mean ± SD (*n* = 5). ^∗^*P* < 0.05, ^∗∗^*P* < 0.01, ^∗∗∗^*P* < 0.005 compared with control group. #*P* < 0.05, ##*P* < 0.01, ###*P* < 0.005 compared with model group.

Finally, we tested whether MT exerts a therapeutic effect against DEX-induced SIOP. Figure S7a shows the *in vivo* MT posttreatment procedure. We found that even when the first administration of MT was significantly delayed, bone density was still partially restored (Figures 8(a)–8(g)**)**. The results of immunohistochemical staining, western blotting, and qRT-PCR further confirmed that even after the bone was exposed to high doses of DEX, MT protected BMSCs from ferroptosis (Figures 8(h)–8(m), Figures S7b–j). Overall, our results show that MT not only contributes to the prevention of SIOP but also plays a vital therapeutic role.

## 4. Discussion

MT is an effective endogenous antioxidant [24]. A recent report indicated that MT has a protective effect against induction of ferroptosis by traumatic brain injury but that this effect is offset by the loss of ferritin H in neurons [25]. Simultaneously, a study showed that MT can ameliorate hypoxic-ischemic brain injury by activating the Akt/Nrf2/Gpx4 signaling pathway [26]. Some scholars have also studied the relationship between MT and ferroptosis in a diabetic osteoporosis model and found that MT can inhibit high glucose-induced ferroptosis by activating the Nrf2/HO-1 signaling pathway, which can be used to treat type 2 diabetic osteoporosis [23]. These reports show that there is a correlation between MT and ferroptosis.

The pathogenesis of SIOP is complicated [27–29]. BMSCs play a key role in normal bone metabolism and are considered ideal seed cells with the potential to maintain and repair bone tissue [30]. GCs inhibit the growth of BMSCs and are important for the induction of osteoporosis by GCs [31, 32]. Our previous studies (Yang Ning, Li Meng, etc.) found that high concentrations of GC can induce oxidative stress and BMSCs death [33]. Several studies have also indicated that oxidative stress contributes to the pathological process of SIOP and that the TXNIP protein can reduce oxidative stress and reverse the process of bone loss by promoting the MOP pathway [34]. Ferroptosis is caused by the inhibition of GPX4 and system xc^−^, which leads to cystine metabolism inhibition, enhanced lipid peroxidation, and iron metabolism dysfunction, and causes the generation of a large amount of ROS and triggers cell death [13]. In the present study, we confirmed that DEX activates the ferroptosis pathway, leading to downregulation of the expression of related markers, such as GPX4, system xc^−^, and FSP1, in BMSCs. Conversely, the observed increase in ROS levels may be due to increased lipid peroxidation induced by DEX, which causes metabolic disorders involving glutamate and cysteine. These results illustrate that DEX induces ferroptotic changes in BMSCs.

Many studies have indicated that MT is closely related to ferroptosis [23, 25, 26, 35]. Considering that MT inhibits ferroptosis in a variety of tissues and organs, it is speculated that MT may have a beneficial effect in our SIOP model. *In vitro* studies have shown that MT can reduce GC-induced ROS levels and ferroptosis. Through micro-CT and H&E staining analysis, we found that MT increased the amount of cancellous bone and trabecular bone to a certain extent, increased the bone density, narrowed the trabecular space, and improved the trabecular arrangement. Immunohistological staining showed that MT increased the number of cells positive for ferroptosis inverse marker proteins such as GPX4, system xc^−^, and FSP1 in femoral tissue. Furthermore, we found that even after SIOP had developed, the use of MT was still able to effectively reduce the expression of ferroptosis-related proteins in BMSCs and the number of ferroptosis in BMSCs, indicating that MT exerted a certain inhibitory effect against ferroptosis and a therapeutic effect against osteoporosis.

The PI3K/AKT pathway is closely related to a variety of orthopedic diseases. It is an intracellular signal transduction pathway that also responds to extracellular signals to promote cell proliferation, metabolism, and growth [36–38]. The key genes involved in this pathway are PI3K and AKT, the activity of which is mediated by phosphorylation of serine or threonine residues in a series of downstream substrates [39]. mTOR is a serine/threonine protein kinase that belongs to the PI3K-related kinase family [40, 41]. mTOR interacts with other protein molecules to form two different complexes [41, 42]. These complexes perform different functions, such as metabolism, cell growth, cell survival, and autophagy, by interacting with different binding partners [43, 44]. Recent studies have shown that mTOR is closely related to the occurrence and development of ferroptosis [45, 46]. The PI3K/AKT/mTOR signaling axis plays an important role in several physiological and pathological processes, including cell proliferation, differentiation, and functional expression [47, 48]. It has been reported that activation of PI3K can functionally inhibit ferroptosis in cancer cells. Mechanistically, the generation of this resistance requires continuous activation of mTORC1 and induction of sterol regulatory element-binding protein 1 (SREBP1) expression. SREBP1 is an mTORC1-dependent protein that acts as an important central transcription factor to regulate lipid metabolism. Monounsaturated fatty acids can mediate the ferroptosis-inhibiting effect of SREBP1, and this process is mainly regulated by stearoyl-CoA desaturase-1 (SCD1), the transcriptional target of SREBP1. In a PI3K-activated breast cancer xenograft mouse model, ferroptosis inhibition combined with mTORC1 inhibition could make tumors almost completely regress [46]. In our study, we also found that treatment of BMSCs with GCs significantly downregulated the expression of PI3K and its downstream proteins AKT and mTOR, while PI3K agonists inhibited GC-induced ferroptosis.

**Figure 9:**
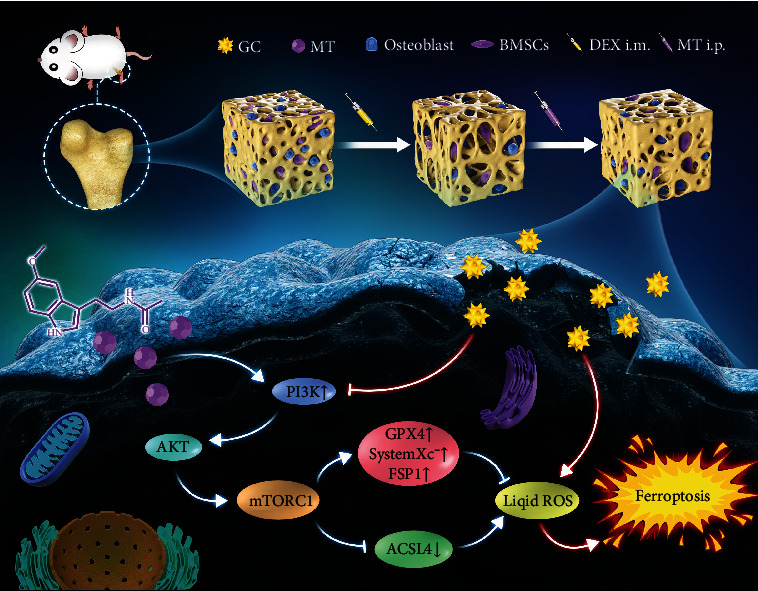
Schematic illustration of MT-mediated protection from GC-induced ferroptosis. Melatonin inhibits the ferroptosis pathway in rat bone marrow mesenchymal stem cells by activating the PI3K/AKT/mTOR signaling axis to attenuate steroid-induced osteoporosis.

Recently, it was reported that MT can alleviate GC-induced osteoblast suppression by activating the PI3K/AKT signaling pathway [49]. Our research also showed that the PI3K pathway was activated by MT. Related studies have shown that human MT1 and MT2 receptors transmit signals through extracellular-regulated protein kinase (ERK)1/2 and that activation of ERK1/2 by these receptors is completely dependent on G protein. The activation of the signaling axis downstream of G protein is the same for the two receptors and involves the activation of the PI3K/PKC*ζ*/c-Raf/MEK/ERK signaling axis [50]. The activation of the ERK pathway is considered to be a marker of ferroptosis, and the level of phosphorylated ERK is positively correlated with the progression of ferroptosis [51]. To more precisely assess the effects of MT on pathways, the PI3K inhibitor GDC0941 was applied to GC-treated BMSCs. We found that GDC0941 reversed the effect of MT, further confirming that MT functions by activating the PI3K pathway. Certainly, further research is needed to determine whether ERK1/2 phosphorylation plays a role in the MT-mediated regulation of PI3K expression. Although our experiments demonstrate that MT has protective from GC-induced ferroptosis, and inhibits the ferroptosis pathway in rat bone marrow mesenchymal stem cells by activating the PI3K/AKT/mTOR signaling axis to attenuate steroid-induced osteoporosis (Figure 9), we should also focus on the contribution and balance between the therapeutic effect of melatonin and bone tissue regeneration, and in addition to the role of the PI3K/AKT/mTOR signaling pathway, other signaling pathways and autophagy in GC-induced SIOP cannot be ignored and should be explored in the future.

## 5. Conclusion

In conclusion, our research confirms that GC-induced ferroptosis is closely related to SIOP. MT can inhibit ferroptosis by activating the PI3K/AKT/mTOR signaling pathway, thereby inhibiting the occurrence of SIOP. Our results indicate that MT may have important implications for the prevention and treatment of SIOP. However, further studies are required to verify our findings.

## Data Availability

All data generated or analyzed during this study are included in this published article. The datasets analyzed during the study are available from the corresponding author on reasonable request.

## Abbreviations

SIOP: Steroid-induced osteoporosis
BMSCs: Bone marrow mesenchymal stem cells
GCs: Glucocorticoids
MT: Melatonin
DEX: Dexamethasone
PI3K: Phosphatidylinositol 3-kinase
AKT: Protein kinase B
mTOR: Mammalian target of rapamycin
ROS: Reactive oxygen species
mtDNA: Mitochondrial DNA
SOD: Superoxide dismutase
GPX: Glutathione peroxidase
SD: Sprague-Dawley
EDTA: Ethylenediaminetetraacetic acid
FBS: Fetal bovine serum
GAPDH: Glyceraldehyde phosphate dehydrogenase
RIPA: Radioimmunoprecipitation assay
SREBP1: Sterol regulatory element-binding protein 1
SCD1: Stearoyl-CoA desaturase-1.

## Supplementary Materials

Supplementary Materials We validated our experimental results and conclusions using additional methods, which are included in the results. Additionally, all results are presented in the supplementary material in the form of figures. We declare that the supplementary material may be co-published with the article.
