# Supplementary material for: Melatonin Inhibits the Ferroptosis Pathway in Rat Bone Marrow Mesenchymal Stem Cells by Activating the PI3K/AKT/mTOR Signaling Axis to Attenuate Steroid-Induced Osteoporosis
Source: Oxid Med Cell Longev. 2022 Aug 18;2022:8223737. doi: 10.1155/2022/8223737 (PMC9410838; doi:10.1155/2022/8223737)
Supplement: Supplementary Materials — We validated our experimental results and conclusions using additional methods, which are included in the results. Additionally, all results are presented in the supplementary material in the form of figures. We declare that the supplementary material may be co-published with the article. [file 8223737.f1.docx]

**Title Page**

**Title:**

Melatonin inhibits the ferroptosis pathway in rat bone marrow mesenchymal stem cells by activating the PI3K/AKT/mTOR signaling axis to attenuate steroid-induced osteoporosis

**The name(s) of the author(s):**

Meng Li^1,†^; Ning Yang^1,†^; Li Hao^2,†^; Wei Zhou^1^; Lei Li^1^; Lei Liu^1^; Fang Yang^3^; Lei Xu^1^; Gang Yao^1^; Chen Zhu^1,^***; Wei Xu^1,^***; Shiyuan Fang^1,^***

**Name and address of the institution**:

1: Department of Orthopaedics, The First Affiliated Hospital of USTC, Division of Life Sciences and Medicine, University of Science and Technology of China, Lujiang Road No. 17, Hefei 230001, Anhui, China.

2: Department of Oncology, The First Affiliated Hospital of USTC, Division of Life Science and Medicine, University of Science and Technology of China, Lujiang Road No. 17, Hefei 230001, Anhui, China.

3: Department of Obstetrics and Gynecology, The Second Affiliated Hospital of Anhui Medical University, Hefei, 230022, Anhui, China.

^†^**These authors contributed equally to this work.**

**Correspondence and requests for materials should be addressed to**

zhuchenustc@163.com (C. Zhu), Tel: +852-62283952, Fax: +852-62283952;

xuweicsgk@sina.com (W. Xu), Tel: +852-62286051, Fax: +852-62286051;

fangsyustc@163.com (S. Fang), Tel: +86-511-62286051, Fax: +86-511-62286051;

**Supplementary data**


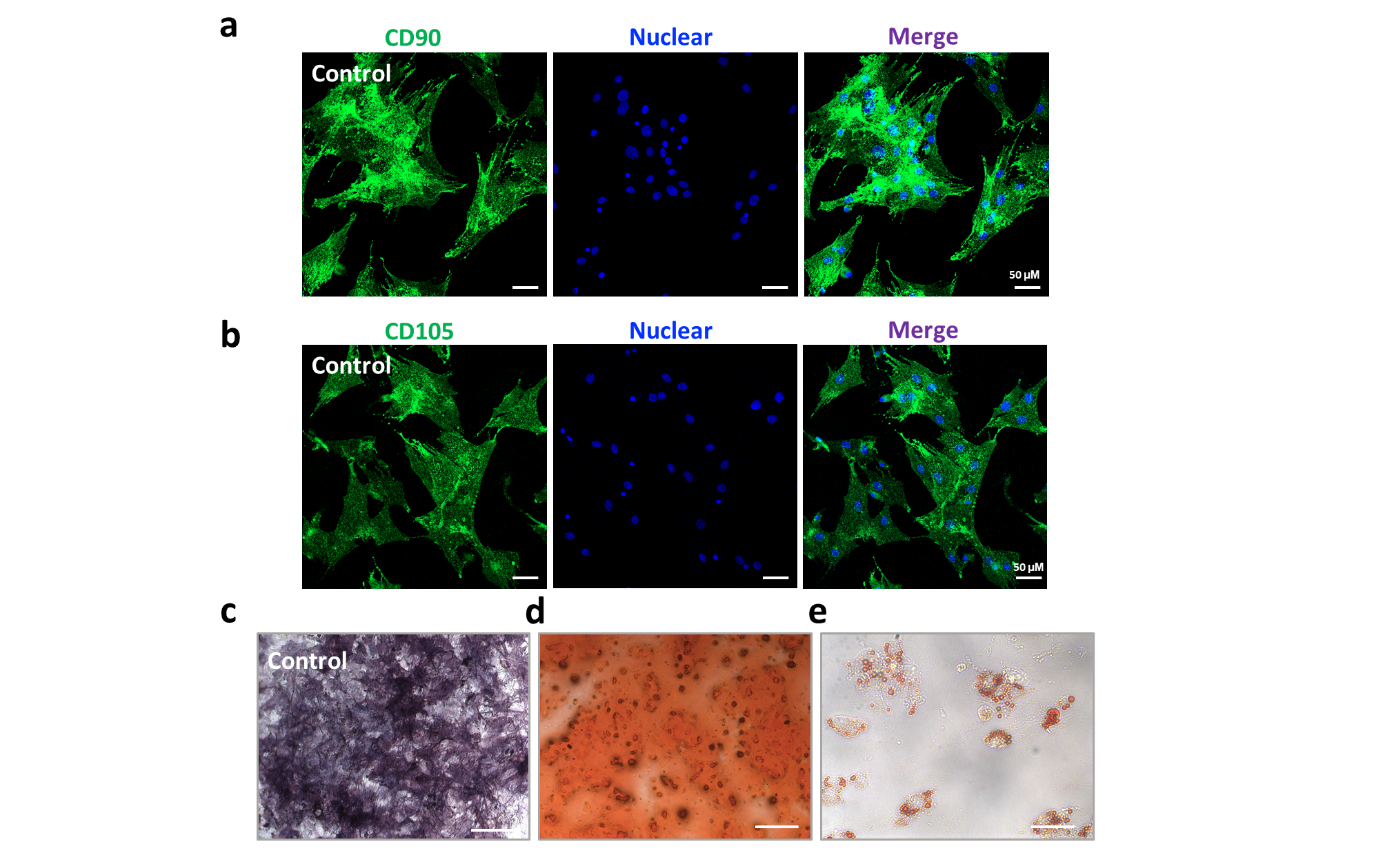


***FigureS1:*** *(a) Images of immunofluorescence staining of CD90 in BMSCs. (b) Images of immunofluorescence staining of CD105 in BMSCs. (c) ALP staining of BMSCs after 7 days of osteogenic induction (Scale bar = 100 μm). (d) ARS staining of BMSCs after 21 days of osteogenic induction (Scale bar = 100 μm). (e) Oil red O staining of BMSCs after 14 days of adipogenic induction (Scale bar = 100 μm). These studies were performed at least 3 biological replicates.*


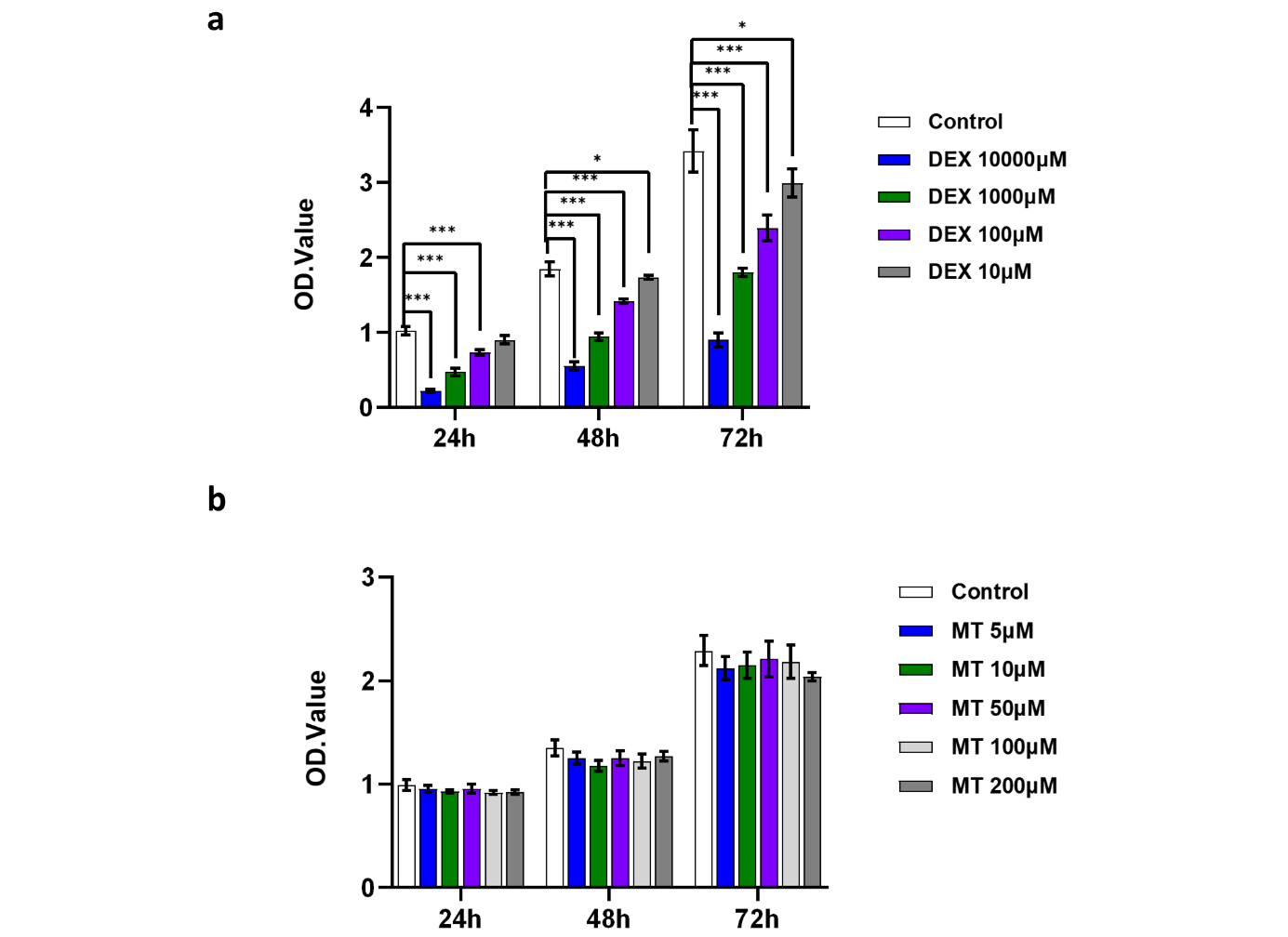


***FigureS2: Cell proliferation and toxicity assay.*** *(a) Cytotoxicity of DEX was assessed on BMSCs using CCK-8 assay. (b) Cytotoxicity of MT was assessed on BMSCs using CCK-8 assay. These studies were performed at least 3 biological replicates. Data represent mean ± S.D. (n=3).*P<0.05, **P<0.01, ***P<0.005 compared with control group.*


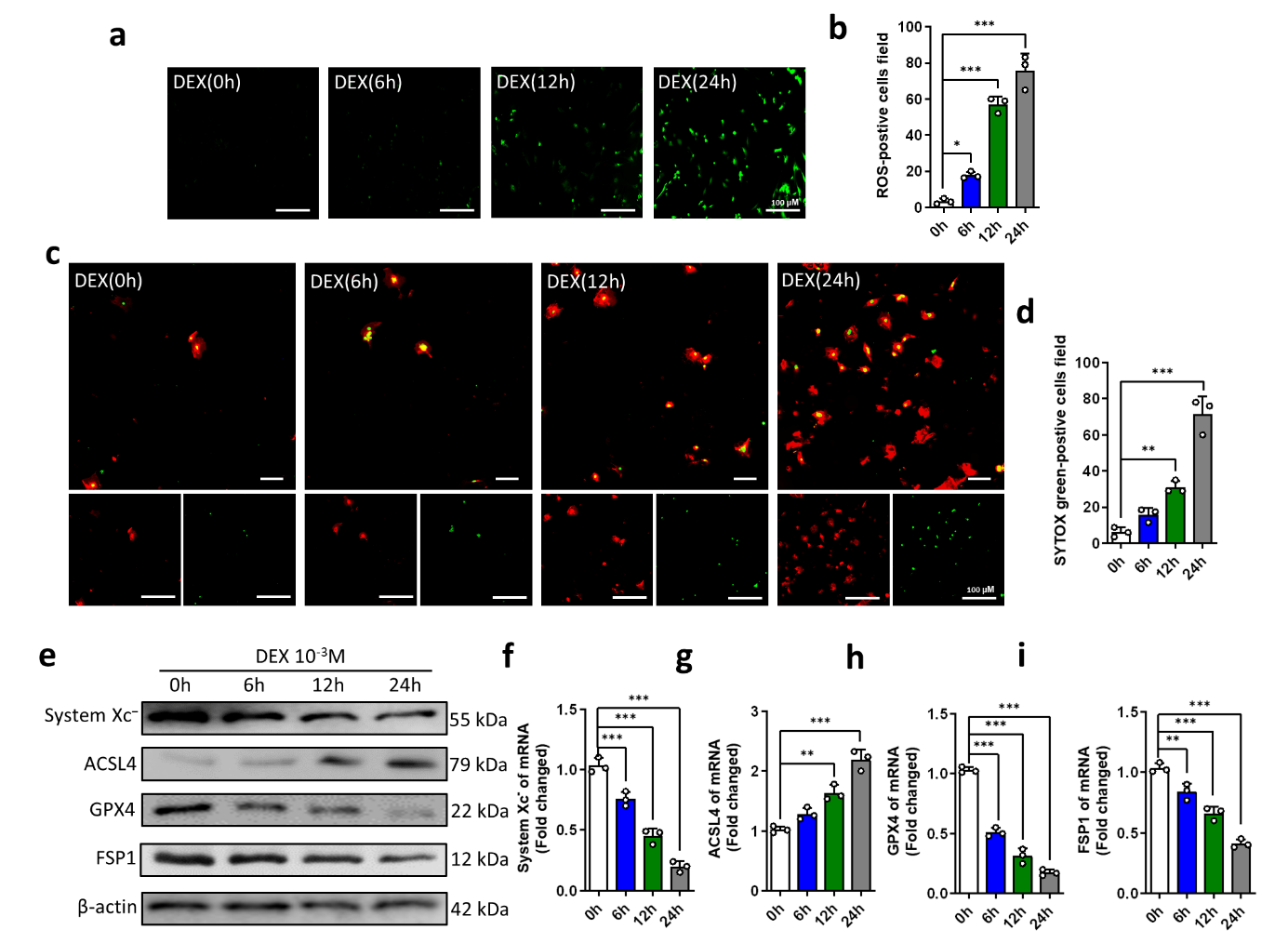


***FigureS3: DEX activates the ferroptotic pathway of BMSCs over time.*** *(a) ROS staining was performed to test the level of oxidative stress in different groups over time. (b) Quantitative analysis of the number of ROS-positive cells per field in (a). (c) Annexin V-mCherry/SYTOX Green detection kit was used to detect cell death. (d) Quantitative analysis of the percentage of SYTOX green-positive cells in (c). (e-i) BMSCs were stimulated with DEX(10^-3^M) for 0h，6h，12h or 24h, and the expressions of system xc^-^, ACSL4, GPX4, and FSP1were analyzed by western blot and qRT-PCR. These studies were performed at least 3 biological replicates. Data represent mean ± S.D. (n=3).*P<0.05, **P<0.01, ***P<0.005 compared with 0h group.*


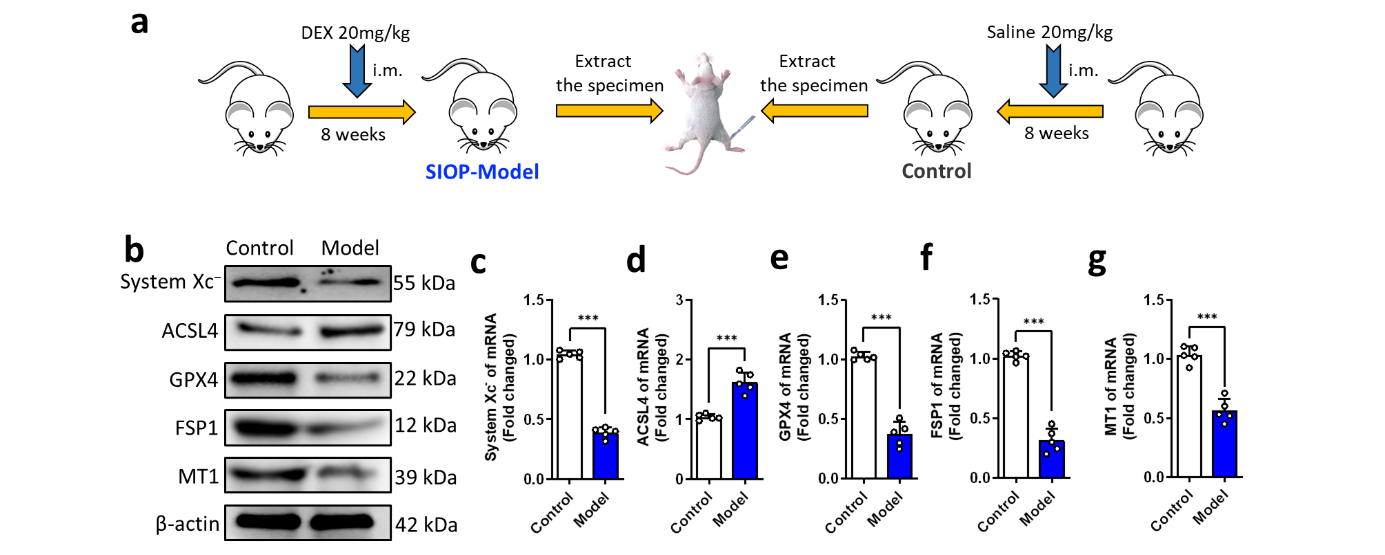


***FigureS4: DEX activates the ferroptotic pathway in SIOP.*** *(a) The timeline of the GC-induced SIOP model. (b-g) The expressions of system xc-, ACSL4, GPX4, FSP1 and MT1 were analyzed by western blot and qRT-PCR. These studies were performed at least 3 biological replicates. Data represent mean ± S.D. (n=5).*P<0.05, **P<0.01, ***P<0.005 compared with control group.*


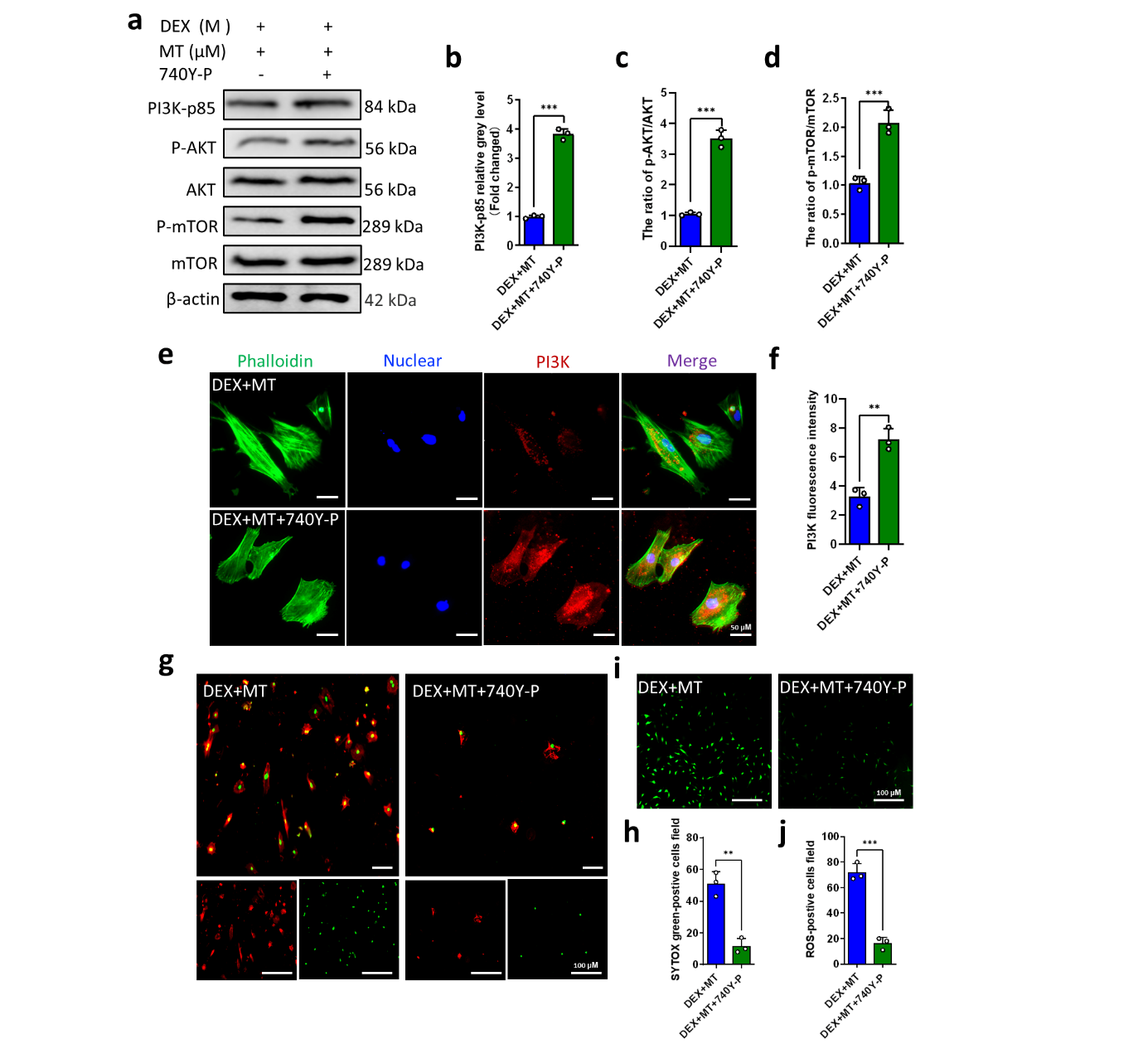


***FigureS5: The positive regulatory effect of MT was significantly enhanced with the use of PI3K agonist.*** *(a-d) Western blot results for the expressions of PI3K-p85, p-AKT, AKT, p-mTOR and mTOR were pretreated with 740Y-P and MT for 24 h; DEX(10^-3^M) was then added for 24 h. (e) Images of immunofluorescence staining of PI3K in BMSCs. (f) Quantification of the fluorescence intensity of PI3K immunofluorescence positively stained cells. (g) Annexin V-mCherry/SYTOX Green detection kit was used to detect cell death. (h) Quantitative analysis of the percentage of SYTOX green-positive cells in (g). (i) ROS staining was performed to test the level of oxidative stress in DEX+MT group and DEX+MT+740Y-P group. (j) Quantitative analysis of the number of ROS-positive cells per field in (i). These studies were performed at least 3 biological replicates. Data represent mean ± S.D. (n=3).*P<0.05, **P<0.01, ***P<0.005 compared with DEX+MT group.*


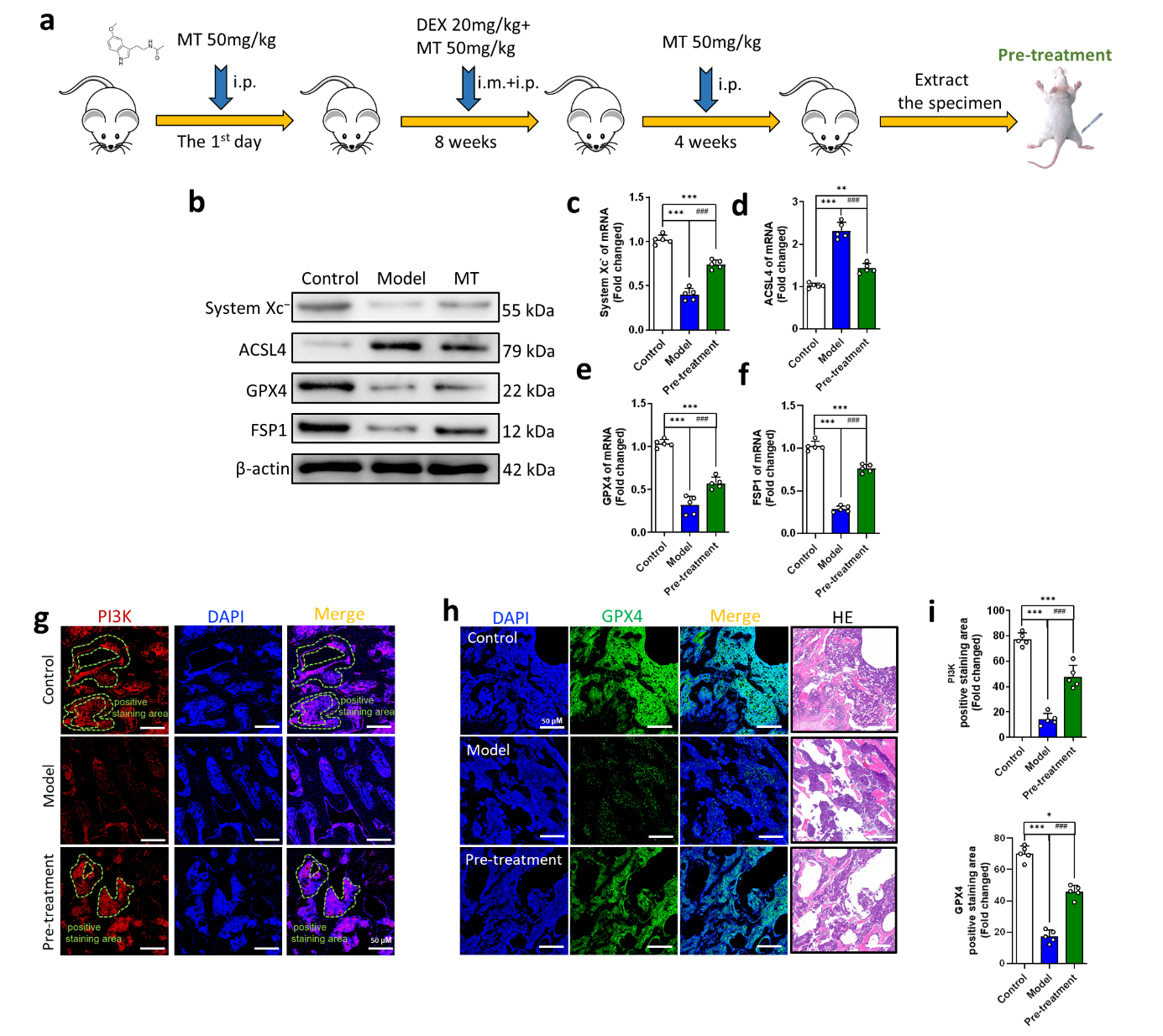


***FigureS6: Pre-treatment with MT alleviates DEX-induced SIOP in vivo.*** *(a) The timeline of the DEX-induced SIOP model and administration of MT in vivo. (b-f) The expressions of system xc-, ACSL4, GPX4 and FSP1 were analyzed by western blot and qRT-PCR in bone tissues between model and Pre-treatment groups. (g) Images of immunofluorescence staining of PI3K in bone tissues. (h) The HE counterstain after the immunofluorescence staining of GPX4 in bone tissues. (i) Quantitative analysis of the area of PI3K-positive stains in (g). (j) Quantitative analysis of the area of GPX4-positive stains in (h). These studies were performed at least 3 biological replicates. Data represent mean ± S.D. (n=5).*P<0.05, **P<0.01, ***P<0.005 compared with control group. ＃P<0.05, ＃＃P<0.01, ＃＃＃P<0.005 compared with model group.*


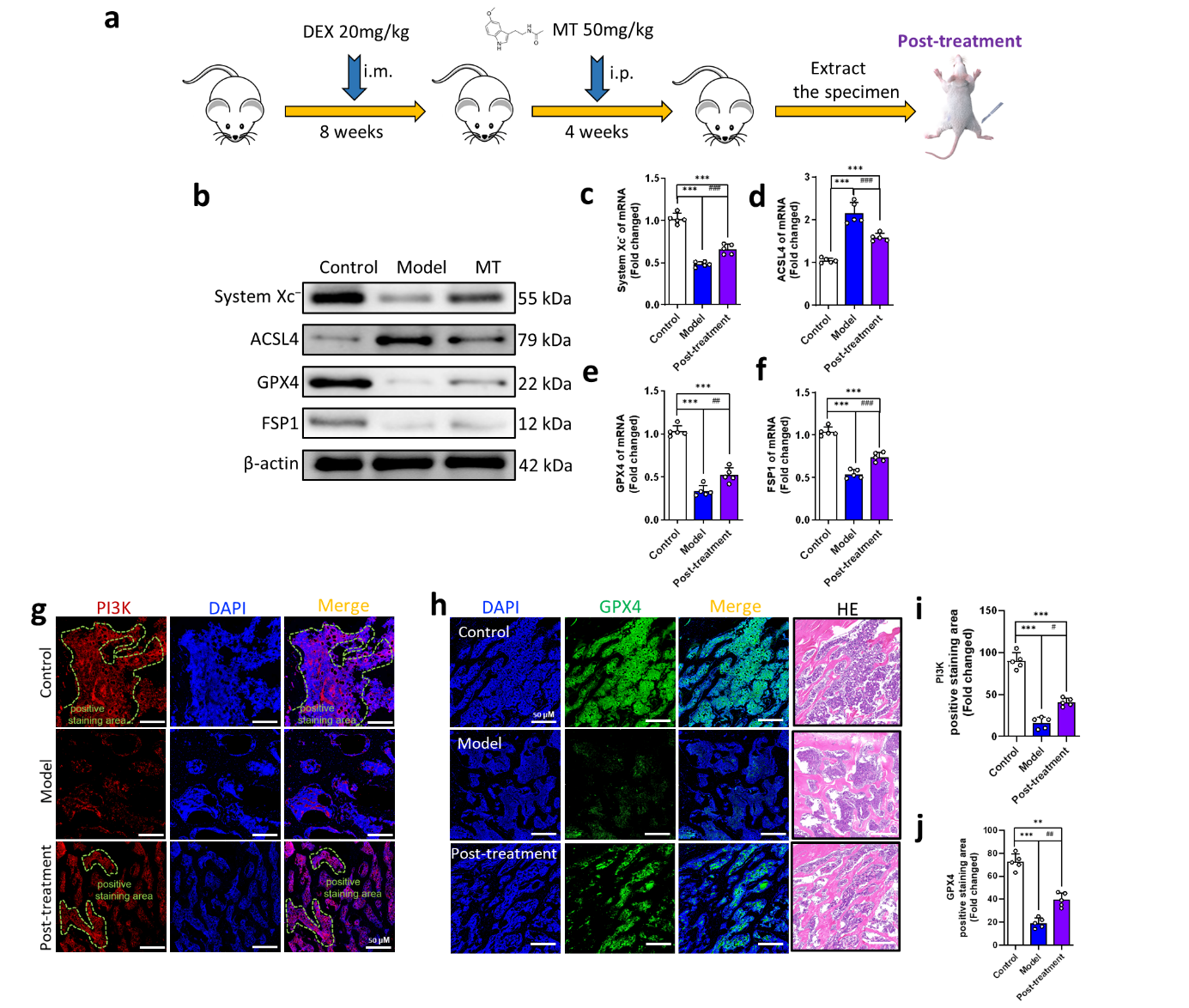


***FigureS7: Even after DEX-induced ferroptotic effects begin, MT can still improve SIOP.*** *(a) The timeline of the DEX-induced SIOP model and administration of MT in vivo. (b-f) The expressions of system xc-, ACSL4, GPX4 and FSP1 were analyzed by western blot and qRT-PCR in bone tissues between model and Pre-treatment groups. (g) Images of immunofluorescence staining of PI3K in bone tissues. (h) The HE counterstain after the immunofluorescence staining of GPX4 in bone tissues. (i) Quantitative analysis of the area of PI3K-positive stains in (g). (j) Quantitative analysis of the area of GPX4-positive stains in (h). These studies were performed at least 3 biological replicates. Data represent mean ± S.D. (n=5).*P<0.05, **P<0.01, ***P<0.005 compared with control group. ＃P<0.05, ＃＃P<0.01, ＃＃＃P<0.005 compared with model group.*
